# The immune response of the whitefly *Trialeurodes vaporariorum* (Hemiptera: Aleyrodidae) when parasitized by *Eretmocerus eremicus* (Hymenoptera: Aphelinidae)

**DOI:** 10.1371/journal.pone.0296157

**Published:** 2023-12-21

**Authors:** Jorge Contreras-Garduño, Pedro Torres-Enciso, Ricardo Ramirez-Romero

**Affiliations:** 1 Laboratorio de Ecología Evolutiva, ENES, Unidad Morelia, UNAM, Morelia, Michoacán, México; 2 Laboratorio de Control Biológico (Lab CB-AIFEN), Departamento de Producción Agrícola, CUCBA, Universidad de Guadalajara, Zapopan, Jalisco, México; Uppsala University, SWEDEN

## Abstract

In insects, the innate immune system is subdivided into cellular and humoral defenses. When parasitoids attack insects, both reactions can be activated and notably, the phenoloxidase (PO) cascade and lytic activity are part of both cellular and humoral defenses. However, to our knowledge, no study has characterized any immune response of the whitefly *Trialeurodes vaporariorum* (Hemiptera: Aleyrodidae) to the attack of *Eretmocerus eremicus* (Hymenoptera: Aphelinidae). Therefore, the first objective of the present study was to determine whether whitefly nymphs recently parasitized by *E*. *eremicus* exhibit any immune response. For this, we estimate the level of prophenoloxidase (proPO), phenoloxidase (PO), and lytic activity by colorimetric assays. A second objective was to assess whether the observed whitefly immune response could be related to a previously reported preference of the predator *Geocoris punctipes* (Hemiptera: Lygaeidae) for non-parasitized nymphs. We therefore offered non-parasitized and recently parasitized nymphs to the predator. Our results show that parasitism of whitefly nymphs by *E*. *eremicus* induced a highly estimated level of proPO and PO, and a lower level of lytic activity. In addition, we found that *G*. *punctipes* did not show a preference for non-parasitized over recently parasitized nymphs. The nymphs of *T*. *vaporariorum* activated the PO pathway against *E*. *eremicus*; however, the increase in proPO and PO levels was traded-off with decreased lytic activity. In addition, the previously reported preference for non-parasitized nymphs was not seen in our experiments, indicating that the induced immune response did not affect predator behavior by *G*. *punctipes*.

## Introduction

The immune system is a hierarchical and modular network of molecules, cells, and tissues that interact to maintain the integrity of individuals [1]. In insects, the innate immune system is subdivided into cellular and humoral defenses [2]. Cellular defense is carried out by hemocytes and fat body cells to produce encapsulation, nodulation, phagocytosis, and cell membrane attack by antimicrobial peptides [3–6]. On the other hand, humoral defense includes the immune deficiency (IMD) and Toll pathways, which produce antimicrobial peptides, the prophenoloxidase pathway, which produces melanin, and the Duox pathway, which favors production of reactive nitrogen or oxygen species [2–4, 7, 8]. When microbial pathogens (e.g., bacteria) invade an insect, the immune response can lead to phagocytosis, the synthesis of antimicrobial substances, or melanotic responses [9]. However, when insects are attacked by macropathogens (e.g., parasitoids), the cellular and humoral phenoloxidase responses are activated [10–12]. Pro-phenoloxidase (proPO) and phenoloxidase (PO) are two main components in the cascade that leads to melanization of the parasite [11]. Lytic activity (LA) includes the production of toxic molecules such as antimicrobial peptides, lysozyme, and lytic enzymes, which leads to the degradation of the invading bodies [7, 13–15]. Parasitoids are insects that spend a phase of their development feeding on another insect (referred to as the ‘host’), which is frequently killed [16]. During the invasion of the host by the parasitoid, the host can produce hemocytes to isolate and destroy the parasitoid [11, 17, 18]. Hemocytes vary between insect host species but include granulocytes, plasmatocytes, phagocytes, oenocytoids, crystal cells, and phagocytes [2, 19–21]. For its part, the melanogenesis process includes the production of different enzymes and molecules such as phenylalanine, tyrosine, cysteine, and eumelanin [17]. During melanogenesis, after introduction of the pathogen or the parasite, pattern recognition proteins bind to pathogen-associated molecular patterns on the parasite, and serine proteases lead the conversion of the pro-form of the prophenoloxidase-activating enzyme (PRO-proPO-AE) into the active proPO-AE. The active proPO-AE, in turn, cleaves proPO to active PO [22]. PO catalyzes the hydroxylation of tyrosine to L-dihydroxyphenylalanine (L-DOPA) to produce melanin and other cytotoxic molecules such as quinones and free radicals [23–25]. Melanogenesis contributes to the encapsulation of the invading parasitoid [11, 17].

The herbivorous insect *Trialeurodes vaporariorum* Westwood (Hemiptera: Aleyrodidae), generally referred to as ´whitefly,´ is an important pest of vegetables, particularly tomatoes [26, 27]. The parasitoid wasp, *Eretmocerus eremicus* Rose and Zolnerowich (Hymenoptera: Aphelinidae) and the predator *Geocoris punctipes* Say (Hemiptera: Geocoridae) are among the natural enemies of *T*. *vaporariorum* [28, 29]. General aspects of the biology and the parasitism process of *E*. *eremicus* have been previously described [29, 30]. For example, the penetration and development of parasitoid larvae of the genus *Eretmocerus* on whiteflies of the genus *Bemisia* or *Trialeurodes* have been previously described by Gelman *et al*. and Gerling *et al*. [31–34]. However, as far as we know, the immune response of *T*. *vaporariorum* during *E*. *eremicus*’ parasitism has not been studied. To the best of our knowledge, only one study related to whitefly immune system function has been reported [35]. Mahadav *et al*. [35] found that during the parasitism of *Bemisia tabaci* (Gennadius) (Hemiptera: Aleyrodidae) by *Eretmocerus mundus* Mercet (Hymenoptera: Aphelinidae), the induction of several immune response-related genes, including *phenoloxidase*, *tetraspanin D107*, *T-complex protein 1 delta subunit* (*TCP1-delta*), and *apolipophorin*, occurred. Therefore, we could expect that in the biological model of the whitefly *T*. *vaporariorum*, there could be an increase of proPO, PO, and lytic activity in response to parasitism by the wasp *E*. *eremicus*. Hence, the first objective of the present study was to determine whether proPO, PO, and lytic activity are expressed by *T*. *vaporariorum* in response to parasitism by *E*. *eremicus*. We then analyzed recently parasitized whitefly nymphs to determine the levels of proPO, PO, and lytic activity.

Previous studies analyzing the interaction between the whitefly *T*. *vaporariorum* and its two natural enemies (*E*. *eremicus* and *G*. *punctipes*) reported that whitefly nymphs with several days of parasitism can still be attacked by the predator *G*. *punctipes* [36, 37]. However, the predator preferred to attack non-parasitized whitefly nymphs over their parasitized counterparts [37]. A possible explanation for this preference could be related to the immune response activation against the parasitoid, which was detected in the first part of our study. That possibility is supported by multiple findings that some predators can discriminate between healthy and infested prey and prefer healthy prey over prey that has been infected with a pathogen [38–40]. To explore this possibility, the second objective of this study was to determine whether the predator also exhibits a preference for non-parasitized nymphs over recently parasitized nymphs. We expected that non-parasitized whitefly nymphs would be preferred over their recently parasitized counterparts, setting the stage for exploration of the relationship between predator preference and prey immune response [41, 42].

## Materials and methods

The environmental conditions and details of the rearing and maintenance of plants and insects, described briefly below, were similar to those described in detail in previous studies [36, 37, 43].

### Biological material

#### Plants

Tomato plants were used for rearing whiteflies. The employed tomato plants were from commercial seeds (var. Saladette) purchased from La Casa del Hortelano SA de CV. The seeds were sown in plastic seedbeds with 26 cavities using a mixture of black soil and perlite as substrate. Once the seedlings had 2 to 4 true leaves, they were transplanted into plastic pots 9.5 cm high and 13 cm in diameter, which contained black soil and perlite in a 50:50 ratio. The transplanted plants were fertilized every third day with ‘triple 18’ fertilizer (Ultrasol, SQM Comercial de México S.A. de C.V.) at a concentration of 0.8 g / L (p / v). The plants were kept inside compartments protected with organza cloth to prevent the attack of other herbivores, under laboratory conditions (24 ± 3°C, 50 ± 10% RH, and a photoperiod of 14:10 L:D) until their use in the experiments. The plants were used in the experiments and for the rearing of herbivores when they had between five and seven developing leaves [37].

#### Whiteflies

The *T*. *vaporariorum* whiteflies used came from colonies established in the ‘Laboratorio de Control Biológico, Area de Insectos Fitófagos y Entomófagos, del Centro Universitario de Ciencias Biológicas y Agropecuarias de la Universidad de Guadalajara’ (LabCB-CUCBA-UdG), Jalisco, Mexico. The flies are virus-free and were taxonomically verified by a specialist in Aleyrodidae, Vicente E. Carapia Ruíz from the Autonomous University of the State of Morelos. Whitefly colonies were kept in acrylic cages (38 cm x 45 cm x 30 cm). Each cage contained between five and six pots of tomato plants, which were infested with whiteflies. The plants were renewed once they were wilted. The colonies were kept under the following conditions: 24 ± 3°C, 50 ± 10% RH and a photoperiod of 14:10 (L:D).

#### Parasitoids

The *E*. *eremicus* parasitoid wasps used in the experiments were provided by Biobest México S.A. de C.V. (San Isidro Mazatepec, Jalisco, Mexico). These wasps were received in the pupal stage and kept in acrylic cages (29.7 x 30.6 x 29.7 cm). Once adult wasps started to emerge, they were fed *ad libitum* with a honey solution diluted in water (ratio 7: 3 v/v) and provided with tap water. Honey and tap water were replaced every day [36, 37]. The females and males used in the experiments were two to four days old, since it is known that females can mate and lay eggs from the first day of life [44]. It was assumed that the parasitoids used in the experiments were mated, because males and females were placed together since their emergence and until their use in experiments [37]. The conditions in which the wasps were kept were 24 ± 3°C, 50 ± 10% RH, and a photoperiod of 14:10 (L:D).

#### Predators

The *G*. *punctipes* predators were obtained from Organismos Benéficos para la Agricultura S.A. de C.V. (Autlán, Jalisco, Mexico). These were received as 5th stage nymphs. Once the insects arrived at the laboratory, they were placed in polystyrene cages (40 cm x 30 cm x 31 cm) and were fed ad libitum with 5 g of artificial diet, [45] 20 ml of water, and food supplements such as pollen (1.8 g) and sorghum seed (3.4 g) to improve their development [46]. Both the artificial diet and the water were changed daily, while the food supplements were changed once a week [37]. Predator females used in the experiments were 8 to 30 days old, as females require a pre-mating period (two to five days) and a pre-oviposition period of 5.2 days [28]. The predators were kept under laboratory conditions (24 ± 3°C, 50 ± 10% RH, and a photoperiod of 14:10, L:D) until their use in experiments [37].

#### Immune system components

According to preliminary assays, it was determined that due to the small size of the nymphs, the most appropriate procedure to analyze proPO, PO, and lytic activity was to gather different numbers of nymphs and perform the analysis of immunological components by grouping individuals, as has been done for other small insects [35, 47]. To assess whether the parasitism of whitefly nymphs elicits an immune response in the nymphs, we analyzed nine treatments that were replicated 13 times each and are described in detail in Table 1.

**Table 1:** Experimental treatments to determine whether the parasitism of *Trialeurodes vaporariorum* nymphs by *Eretmocerus eremicus* elicits an immune response in the whitefly nymphs.

Treatment	Number of individuals per replicate	*T*. *vaporariorum* stage	Parasitism Status	Replicates	Total number of analyzed individuals
**PBS** (Negative control)	None	None	None	13	None
***Tm*** (Positive control)	1	None	None	13	13
***50 Tv***	50	Nymphs	Non-parasitized	13	650
***100 Tv***	100	Nymphs	Non-parasitized	13	1300
***150 Tv***	150	Nymphs	Non-parasitized	13	1950
***50 Parasitized-Tv***	50	Nymphs	Parasitized	13	650
***150 Parasitized-Tv***	150	Nymphs	Parasitized	13	1950
***50 Tv-Adults***	50	Adults	Non-parasitized	13	650
***100 Tv-Adults***	100	Adults	Non-parasitized	13	1300

**PBS**: PBS solution only; ***Tm***: *T*. *mollitor* haemolymph from larvae previously infected with *M*. *anisoplae*; ***Tv***: *Trialeurodes vaporariorum*.

We followed previously validated procedures to determine proPO, PO, and AL [14, 15, 48–51], described below. We analyzed three different groups of non-parasitized *T*. *vaporariorum* nymphs, two groups of parasitized nymphs, two groups of whitefly adults, and two control groups. The three groups of non-parasitized nymphs (hereinafter referred to as ‘*Tv*’) were prepared with 50, 100, and 150 whitefly nymphs (2nd and 3rd instar) per replicate. Each whitefly nymph group was prepared by carefully removing nymphs from tomato leaflets and placing them together in Eppendorf® tubes (1.5 ml) containing 100 μl of phosphate buffer 0.1 M (K_2_HPO_4_-KH_2_PO_4_ [Caiman Chemical Co]) (hereinafter referred to as PBS) per tube. The two groups of parasitized *T*. *vaporariorum* nymphs (hereinafter referred to as ‘*Parasitized-Tv*’) were composed of 50 and 150 parasitized whitefly nymphs. To obtain these parasitized nymphs, leaflets bearing 60 to 100 second and third instar whitefly nymphs were placed in Petri dishes (9 cm diameter) where 10 pairs of *E*. *eremicus* wasps (2 to 3 days old) were introduced and allowed to parasitize over the course of 48 h. The number of wasps introduced was chosen in accordance with the daily rate of oviposition of *E*. *eremicus* and host density on the leaflets [29]. After this exposure period, adult wasps were removed from the Petri dishes and the parasitized nymphs were allowed to develop for 7 days. On the seventh day after the extraction of the wasps, the nymphs were carefully collected from the leaflets and gathered in Eppendorf® tubes (1.5 ml) containing 100 μl of PBS per tube to form the two groups of 50 and 150 parasitized nymphs. Parasitized nymphs were sampled on the seventh day after exposure to the parasitoid because in preliminary observations, we determined that under our experimental conditions, *E*. *eremicus* larvae penetrate the *T*. *vaporariorum* nymphs 6 to 7 days after egg oviposition.

The groups containing whitefly adults were prepared by sampling adults from whitefly cohorts. Sampled adults were placed together to form groups containing 50 and 100 adults per Eppendorf® tube (1.5 ml) containing 100 μl of PBS each. Finally, the negative control consisted only of the employed diluent (i.e., 100 μl of PBS) and the positive control consisted of *Tenebrio molitor* (Coleoptera: Tenebrionidae) hemolymph (50 μl) obtained from larvae that were previously infected with *Metarizhium anisoplae* (Hypocreales: Ascomycota). Previous analyzes of sub-samples of that hemolymph read positive for the presence of proPO, PO, and lytic activity. The employed process to infect *T*. *molitor* larvae with *M*. *anisoplae* and to obtain the hemolymph samples was the standard one described in detail by Medina-Gómez *et al*. 2018 [52]. Samples followed standard preparation procedures as described in detail by Méndez-López *et al*. 2021 [53]. Overall, samples from each tube were macerated with sterile tips in 100 μL of PBS. Then, they were centrifuged at 5200 g at 4°C for 5 minutes, and 70 μL of supernatant was transferred to new tubes containing 100 μL of PBS. These sample tubes were stored in a CryoCube® F570 freezer at -70°C. From there, the different amounts necessary for the analysis of the immunological components (i.e., proPO, PO, and lytic activity) were obtained from these sample tubes as described below. Each treatment was replicated 13 times, so in total, 3,900 non-parasitized whitefly nymphs, 2,600 parasitized whitefly nymphs, and 1950 whitefly adults were prepared and analyzed.

To estimate proPO, in each of a 96-well ELISA plate (CorningTM), 38 μL of PBS, 50 μL of the sample, 2 μL of cetylpyridium chloride (after this referred to as CPCH) (Sigma; 2.5 mg in 1 mL of PBS) and 10 μL of L-dihydroxyphenylalanine (from now on referred to as L-DOPA) (Sigma; 0.4 mg in 1 mL of PBS) were added. We vortexed the solution of L-DOPA for 30 min until dissolved while being protected from light in aluminum foil and then cooled at 4° C. The mixture of L-DOPA, CPCH, and samples was allowed to incubate at room temperature for 30 minutes and then read at 490 nm for 30 minutes with an ELISA reader (Microplate Reader Series, BMG LABTECH). Negative (only PBS with L-Dopa) and positive (hemolymph of *T*. *molitor* infected with *M*. *anisopliae*, L-Dopa, and PBS) controls were included in each plate. It is important to note that this procedure allows us to estimate how much proPO was present because proPO has a zymogen that inhibits its activity. Adding L-Dopa, we dissociate, activate, and detect the enzyme. In the case of PO, a method like that used for proPO was employed, but for PO, CPCH was not added [14, 15]. We decided to use CPCH because we get more activity in our pilot experiments and standardizations than using α-chymotrypsin. In both proPO and PO, the mean absorbance reading was calculated for each treatment, with higher absorbance averages indicating more significant enzymatic activity. In addition, for each treatment, linear regression curves and the corresponding formulas were estimated to appreciate the enzymes’ kinetics better. The incubation time of the mixture before reading in ELISA may vary depending on the species [54, 55]. We decided to incubate the samples for 30 minutes because we observed very low activity of these enzymes during that period in our standardizations.

To estimate the lytic activity, in each well of the 96-well ELISA plate (CorningTM), 30 μL of sample and 200 μL of *Micrococcus lysodeikticus* solution (Sigma; 7.2 mg / 20 mL PBS) were added. Negative (PBS and *M*. *lysodeikticus* solution) and positive (*T*. *molitor* hemolymph infected with *M*. *anisoplae* and *M*. *lysodeikticus* solution) controls were included in each plate. The plate was allowed to incubate for 30 minutes at room temperature and then it was read at 540 nm for 30 minutes. The lytic activity was analyzed via absorbance readings relative to those of the controls. For each treatment, the average absorbance reading was calculated, as well as the linear regression curves and the corresponding formulas. Here, the lower readings and slopes would denote greater activity because they refer to the elimination of *M*. *lysodeikticus* due to a higher lytic activity [15].

### Predator’s preference

To determine the predator’s preference for early parasitized or non-parasitized nymphs, we followed a randomized block design. The fixed factor was the ‘parasitism status’ (two levels: parasitized and non-parasitized) and ‘time’ was considered as the blocking factor. As a result, the two treatments (parasitized and non-parasitized nymphs) were established simultaneously at each arena (choice bioassay). The recorded response variable was the number of nymphs preyed upon by the predator at each treatment. The procedure for obtaining parasitized and non-parasitized nymphs was the same as described in the previous section. Parasitized nymphs used in this experiment were 7 days post-parasitoid exposure. We consider these parasitized nymphs as being ‘early parasitized’ taking into account that parasitoids take about 22 days post-parasitoid exposure to emerge as adults [37]. Arenas were established as described in Velasco-Hernández *et al*. [37]. Overall, each arena consisted of a petri dish (9 cm diameter) with an agar layer (5 mm thickness) that contained 18 non-parasitized and 18 parasitized nymphs, randomly distributed in the arena surface. When arenas were set up, a predator female (previously starved for 24 hours) was released into the arena and allowed to predate during 24 hours. The number of preyed-upon nymphs and their status (parasitized or not) was recorded after 6 and 24 hours following the release of the female predator [43]. Each arena was replicated 14 times and for each replicate, new plants and insects were employed to avoid pseudo-replication.

## Statistical analysis

For the analysis of the response variables, the assumptions of normality and homoscedasticity were first tested [56]. If the data met the assumptions, an analysis of variance (ANOVA) was performed through a simple linear model. When the assumptions were not met, the best model was chosen using the Akaike Information Criterion (AIC) [57]. Based on the above, the response variables mean absorbance of proPO and lytic activity were compared among treatments through a one-way ANOVA, after the data were transformed with log (*x* + 1) and sqrt (*x* + 0.05), respectively. In both cases, the treatment was the fixed factor and the block was integrated to the models as a random factor. The mean absorbance for PO was compared among treatments through a mixed linear model (LMM) [58] using the treatment as a fixed factor and the block as the random factor.

The mean number of preyed-upon nymphs at 6 and 24 hours was compared between parasitized and non-parasitized nymphs using a Student’s *t*-test, since the data met the assumptions of homoscedasticity [56]. The linear regression curves of proPO, PO, and LA treatments and the formulas for each curve were obtained using the *ggplot2* and *dplyr* R-packages. All analyses were performed using the software R, version 4.0.1. [59].

## Results

### proPO and PO analysis

**Fig 1:**
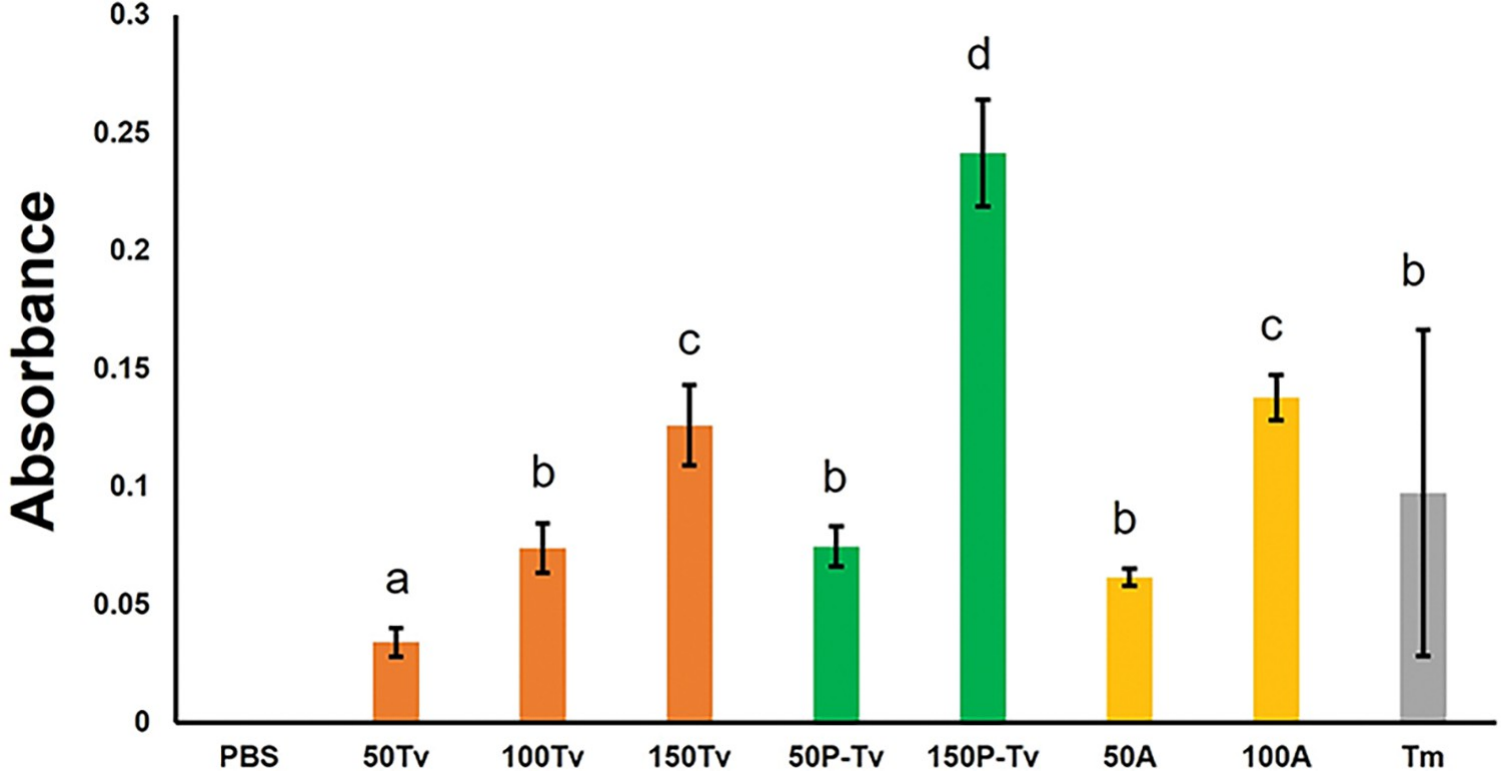
Mean absorbance levels of activated prophenoloxidase (proPO) in parasitized and non-parasitized nymphs and adults of the whitefly *T*. *vaporariorum*. PBS (= negative control). 50Tv, 100Tv, and 150Tv (= 50, 100, and 150 non-parasitized *T*. *vaporariorum* nymphs, respectively). 50P-Tv and 150P-Tv (= 50 and 150 parasitized *T*. *vaporariorum* nymphs, respectively). 50A and 100A (= 50 and 100 whitefly adults, respectively). Tm (= positive control: hemolymph of *T*. *molitor* infected with the entomopathogenic fungus *M*. *anisoplae*). Columns bearing different letters represent significant differences (at *P* < 0.05).

When parasitized nymphs were compared with their non-parasitized counterparts (Comparisons 50Tv vs 50P-Tv and 150Tv vs 150P-Tv), the parasitized nymphs showed significantly higher proPO estimated levels (*F*_8,86_ = 17.521; *P* < 0.001) (Fig 1).

This result indicates that the parasitism of *T*. *vaporariorum* nymphs by *E*. *eremicus* would induce a higher presence of proPO. We also found that the highest amount of proPO was estimated in the treatment with 150 parasitized nymphs (150P-Tv); the level was even higher than in the positive control (Tm) (Fig 1). The lowest level of proPO was found in the treatment with 50 non-parasitized nymphs (50Tv) (Fig 1). When similar numbers of non-parasitized nymphs and adults (Comparisons 50Tv vs 50A and 100Tv vs 100A) were compared, higher levels of proPO were estimated with adults. The same trends observed with the means were found with the regression curves, with higher estimated amounts of proPO on parasitized nymphs relative to non-parasitized counterparts (S1 Fig). In addition, tiny slopes and R^2^ values were found, indicating that the absorbance values change little over time with a weak absorbance-time correlation (S1 Fig).

**Fig 2:**
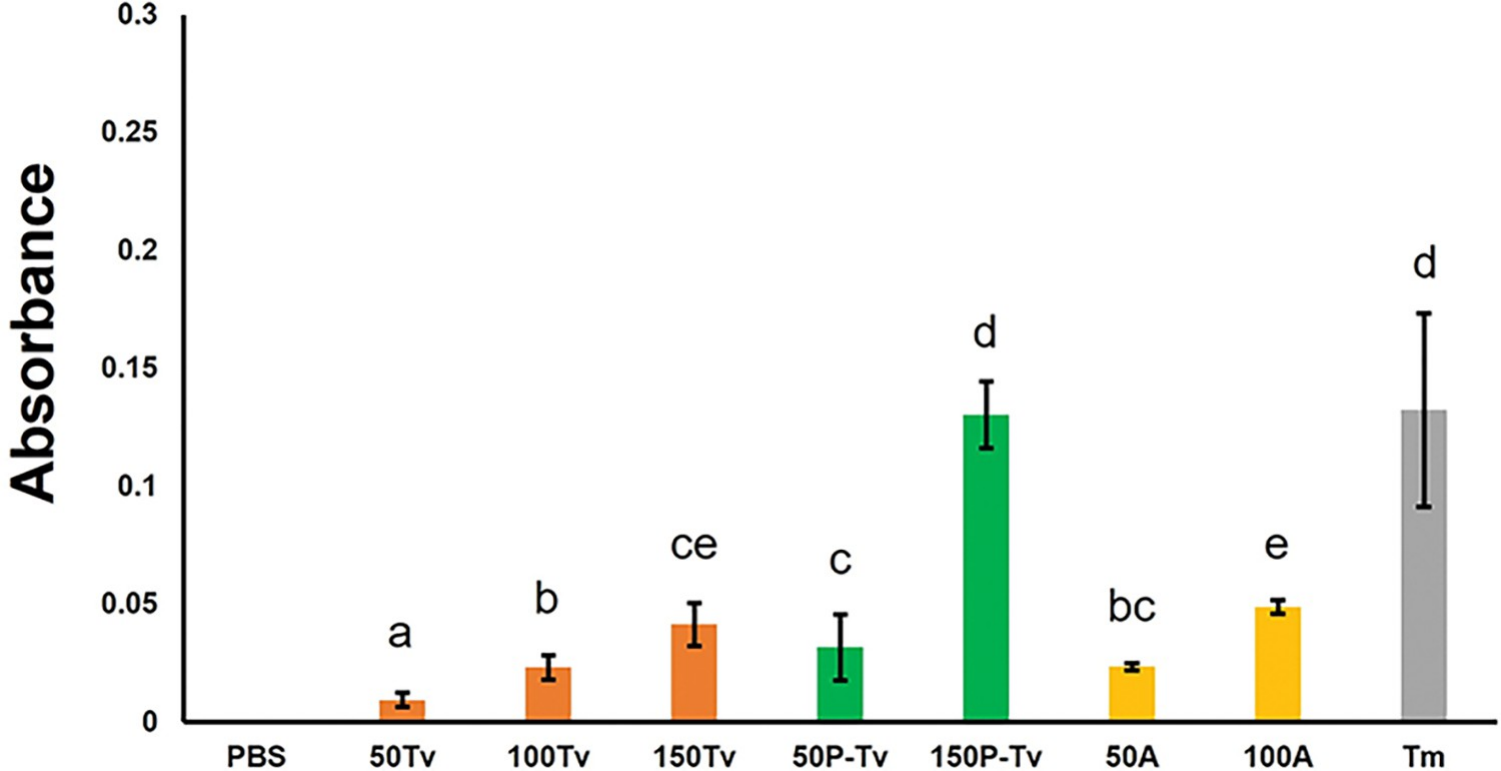
Mean absorbance levels of the enzymatic activity of phenoloxidase (PO) in parasitized and non-parasitized nymphs and adults of the whitefly *T*. *vaporariorum*. PBS (= negative control); 50Tv, 100Tv, and 150Tv (= 50, 100, and 150 non-parasitized *T*. *vaporariorum* nymphs, respectively); 50P-Tv and 150P-Tv (= 50 and 150 parasitized *T*. *vaporariorum* nymphs, respectively); 50A and 100A (= 50 and 100 whitefly adults, respectively); Tm (= positive control: hemolymph of *T*. *molitor* infected with the entomopathogenic fungus *M*. *anisoplae*). Columns bearing different letters represent significant differences (at *P* < 0.05).

As with the proPO estimated levels, the PO levels also showed significant differences among treatments (*F*_8,86_ = 20.658; *P* < 0.001) (Fig 2). Similar to proPO, the expression of PO in the parasitized nymphs was significantly higher than that in their non-parasitized counterparts (comparisons 50Tv vs. 50P-Tv and 150Tv vs 150P-Tv) (Fig 2). Overall, we observed that the highest PO expression was found in the positive control (Tm) and in the treatment with 150 parasitized nymphs (150P-Tv). In contrast, the lowest PO quantity was obtained in the treatment with 50 non-parasitized nymphs (50Tv) (Fig 2). When regression curves were obtained, similar trends were found with parasitized nymphs expressing higher levels of PO than non-parasitized nymphs (S2 Fig). For its part, the values of the slopes and R^2^ were small, also indicating small changes over time and a weak PO activity-time correlation (S2 Fig).

### Lytic activity

**Fig 3:**
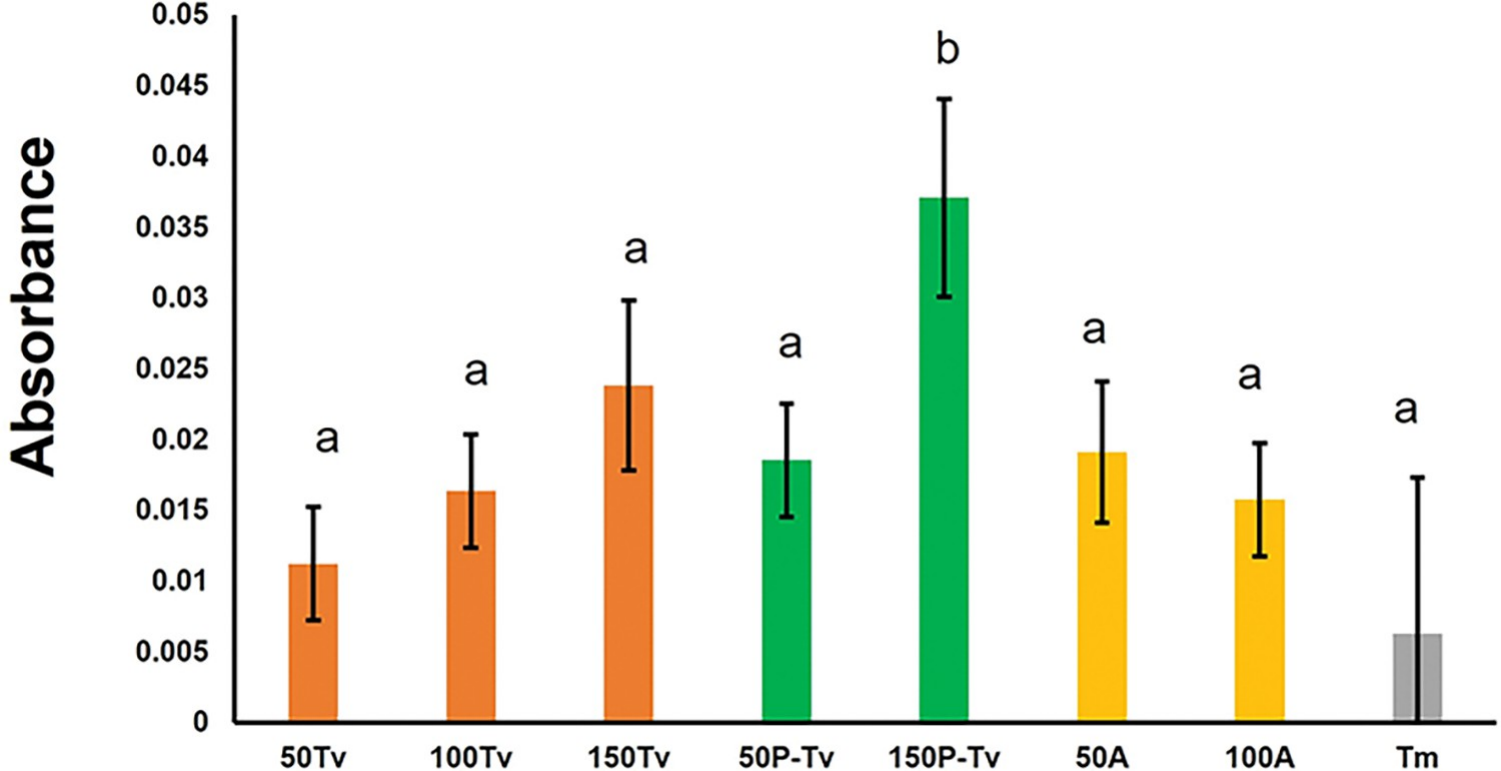
Mean absorbance levels of lytic activity in parasitized and non-parasitized nymphs and adults of the whitefly *T*. *vaporariorum*. 50Tv, 100Tv, and 150Tv (= 50, 100, and 150 non-parasitized *T*. *vaporariorum* whitefly nymphs, respectively). 50P-Tv and 150P-Tv (= 50 and 150 parasitized *T*. *vaporariorum* nymphs, respectively). 50A and 100A (= 50 and 100 whitefly adults, respectively). Tm (= positive control: hemolymph of *T*. *molitor* infected with the entomopathogenic fungus *M*. *anisoplae*). Columns bearing different letters represent significant differences (at *P* < 0.05).

Significantly lower lytic activity was seen only for the treatment with 150 parasitized nymphs (*F*_2,86_ = 3.176; *P* = 0.003) (Fig 3). For this treatment, the absorbance reading was significantly higher than for the other treatments, which indicates lower lytic activity (Fig 3). There were no significant differences among the other treatments, which indicates a similar lytic activity (Fig 3). The regression curves observed this pattern, where the highest absorbance values were obtained for the 150P-Tv group (S3 Fig). Additionally, this group tended to increase absorbance over time (i.e., reduction in lytic activity over time) compared to the other treatments (S3 Fig). Finally, the slopes and R^2^ values were relatively low, indicating little change in lytic activity over time (S3 Fig).

### Predator’s preference

When the mean number of preyed-upon nymphs after 6 hours was compared between parasitized and non-parasitized nymphs, no significant difference was seen (*t* = 0.127; *df* = 25.834; *P* = 0.899). We found that *G*. *punctipes* consumed 13.0 (±1.51) (mean ± SEM) and 13.28 (±1.64) non-parasitized and parasitized nymphs, respectively. Similarly, no significant difference was found between the mean number of non-parasitized and parasitized nymphs preyed upon over 24 hours (*t* = 0.294; *df* = 25.812; *P* = 0.770). The predator consumed 15.21 (±1.63) non-parasitized nymphs and 14.5 (±1.78) parasitized nymphs after 24 hours.

## Discussion

The first objective of the present study was to determine whether *T*. *vaporariorum* exhibits an immune response during the first parasitism phase by *E*. *eremicus*. Therefore, we estimated the proPO, PO levels, and lytic activity in recently parasitized nymphs relative to non-parasitized coequals. We found that the estimated levels of proPO and PO in parasitized nymphs, compared to non-parasitized counterparts, were significantly higher, whereas lytic activity was lower.

On the one hand, proPO is an inactive zymogen precursor of PO, which is a crucial enzyme for humoral (melanization) and cellular (encapsulation) responses against parasites [7, 60, 61]. When parasitoids attack insects, proPO is converted into PO to produce melanin [61–63]. Melanization is critical in killing microorganisms and parasites by hardening the cells surrounding the pathogen to isolate the foreign organism from the rest of the host body [17, 64]. It is also known that proPO activation can result in the formation of melanotic capsules that can kill invaders via reactive oxygen species [65]. Therefore, our results indicate that, in whitefly nymphs responding to parasitism, the recognition system favors the proPO pathway, probably to induce encapsulation and cytotoxic effects such as reactive oxygen species production (a ubiquitous host defense mechanism against parasitoid attack [66]). Future research could shed light on which of the processes is dominant in fighting against the parasitoid larvae: encapsulation and/or melanization.

The present results are the first report that whitefly nymphs produce these immune enzymes in response to attack by parasitoid wasps. Furthermore, they are consistent with the results of Mahadav *et al*. (2008), [35] who found induction of immune response-related genes such as the *phenoloxidase*, *tetraspanin D107*, *TCP1-delta*, and *apolipophorin* genes when *B*. *tabaci* is parasitized by *E*. *mundus*. We also found that non-parasitized nymphs exhibit a baseline, above zero levels of proPO and PO, which is in line with previous reports of prophylactic immunity in insects before infections occur [67, 68].

Lytic activity is related to the action of proteolytic and hydrolytic enzymes responsible for degrading invading pathogens [69]. Lytic activity is carried out by compounds such as antimicrobial peptides and lysozymes, [70] that favor elimination of the parasite after recognition, and exposure of the parasite molecules to initiate the development of adaptive immunity [13]. Thus, when analyzing lytic activity in our results (Fig 3), we found that almost all treatments (except one) exhibited similar levels of lytic activity expression. This result suggests that the immune defense processes linked to lytic activity are not launched for whiteflies attacked by a parasitoid. This possibility is postulated considering that lytic activity has been mainly related to the response against pathogens such as bacteria [69]. Another possible explanation is that lytic activity is complementary to activation of the proPO pathway [71]. Consistently, Rao *et al*. (2010) [72] found in *Manduca sexta* that proPO inhibits lysozyme activity and its interaction with IML-3, a C-type lectin that can adhere to bacteria. Hence, the activation of PO may impair lytic activity. In line with this possibility, we observed that lytic activity in the group of 150 parasitized whiteflies was significantly lower than in the 150 non-parasitized whiteflies group. This same trend was observed when comparing the treatments with 50 nymphs, suggesting that in whitefly nymphs recently parasitized by *E*. *eremicus*, the defense mechanisms rely mainly on the PO cascade and could have a cost over those mechanisms related to lytic activity [73]. When we analyzed the regression curves (enzyme kinetics) of the estimated proPO, PO, and LA, the results generally indicate that the trends found with the mean absorbances are confirmed, with the parasitized nymphs showing higher levels of proPO and PO and less lytic activity.

Additionally, it was found that changes in the levels of these compounds over time were minimal. Yet, it was found that for proPO, PO and LA, the group with the highest slope had 150 parasitized nymphs (150P-Tv). These results suggest that over time, in that group of parasitized nymphs (150P-Tv), the amount of proPO and PO will increase, while LA will decrease over time. These trends reinforce the hypothesis that there could be a trade-off in the production of PO and LA discussed above. Additional studies are needed to test this possibility.

On the other hand, it is essential to note that due to the small size of the nymphs, we decided to gather several individuals to analyze them in groups as in previous studies [35, 47]. We also followed that procedure with the parasitized nymphs that contained parasitoid larvae inside [31–34]. Under this procedure, we assumed that the immune responses detected come from the host, not the parasitoid larvae. This assumption is because it would be counterintuitive to expect the parasitoid larvae to produce the same host’s immune enzymes that it delivers to defend itself against parasitism. From what we know about the parasitoid-host interaction, we would expect that the parasitoid larvae would instead synthesize proteins that inhibit the production of PO, as documented in other studies [74–76]. Future studies are warranted to determine whether the parasitoid produces such proteins to counterattack the immune system of the whitefly in the biological model *E*.*eremicus*-*T*. *vaporariorum*.

Overall, our results can open up new research topics; for example, to determine the costs and benefits of this immune response exhibited by whiteflies in response to parasitism. It is known that the immune response can be costly in terms of growth and reproduction [77]. Therefore, it would be interesting to determine whether the immune response exhibited by whiteflies carries some cost in this sense; for example, whether the immune response is related to lower progeny production or adults exhibiting shorter life spans [78]. On the other hand, future research can focus on studying the process of gene activation and the production of proteins related to the immune response in this biological model using transcriptomics or proteomic approaches [e.g., 79, 80]. Additionally, from an applied point of view, it will be essential to determine to what extent the immune response is related to the survival and emergence rates of the whitefly after exposure to parasitoids [36]. If the whitefly’s immune system plays an essential role in defense against the parasitoid’s attack, it would be interesting to study how weakening the immune response of the whitefly makes this pest more vulnerable to attack by pathogens or its entomophagous [3, 81].

Regarding predator preference, Velasco-Hernández *et al*. (2013) [37] previously reported that the IG-predator *G*. *punctipes* preferred non-parasitized over parasitized nymphs. This preference can be explained by at least two possibilities. First, the hardening of the parasitoid pupa inside the host nymph could make it difficult for the IG-predator to consume the nymphs, resulting in a preference for non-parasitized nymphs [82]. Second, the predator could detect biochemical changes related to the nymph’s immune response against the parasitoid attack, [83] resulting in avoidance of consumption of parasitized nymphs. It is known that some predators avoid consuming infected or parasitized prey [38, 40]. The second objective of our study was therefore to determine whether the assessed whitefly immune response could be related to the previously reported preference of *G*. *punctipes* (IG-predator) for non-parasitized over parasitized nymphs. We expected that the predator would prefer unparasitized nymphs over their recently parasitized counterparts. However, our results did not support that expectation; the predator showed no preference. These results seem to contradict those previously reported by Velasco-Hernández *et al*. [37]. However, it is essential to note that these authors tested parasitized nymphs of advanced age (approx. 23 days post-parasitism), while we used recently parasitized nymphs (approx. 7 days post-parasitism). This suggests that the IG-predator preference is not related to the immune response of the nymph against the parasitoid. Instead, our results support the hypothesis that the preference of the IG-predator is related to the hardening of the parasitoid pupa and difficulty of consumption. Subsequent studies inserting nylon micro-grafts into whitefly nymphs could help to test this alternative explanation.

## Conclusions

In conclusion, our results show that *T*. *vaporariorum* whitefly nymphs activated proPO and PO but exhibited reduced lytic activity against the parasitoid *E*. *eremicus*. Future studies should test why these molecules are negatively correlated. In addition, we found that at the time of nymph parasitism, the predator does not show a preference for non-parasitized vs. recently parasitized nymphs. Thus, it will be necessary to test alternative hypotheses to explain the predator’s preference for non-parasitized over parasitized nymphs of advanced stage of development.

## Supporting information

S1 Fig **Top**: absorbance levels of activated prophenoloxidase (proPO) in parasitized and non-parasitized nymphs and adults of the whitefly *T*. *vaporariorum*. **Bottom:** estimated linear equation and R^2^ value for each treatment, description of abbreviations is also provided.
(TIF)

S2 Fig **Top**: absorbance levels of the enzymatic activity of phenoloxidase (PO) in parasitized and non-parasitized nymphs and adults of the whitefly *T*. *vaporariorum*. **Bottom:** estimated linear equation and R^2^ value for each treatment, description of abbreviations is also provided.
(TIF)

S3 Fig **Top**: absorbance levels of lytic activity (LA) in parasitized and non-parasitized nymphs and adults of the whitefly *T*. *vaporariorum*. **Bottom**: estimated linear equation and R^2^ value for each treatment, description of abbreviations is also provided.
(TIF)

S1 Data Raw data from the absorbance readings recorded in all replicates of the different proPO, PO, and LA treatments. (XLSX)
