# Supplementary figures and images for: The immune response of the whitefly Trialeurodes vaporariorum (Hemiptera: Aleyrodidae) when parasitized by Eretmocerus eremicus (Hymenoptera: Aphelinidae)
Source: PLoS One. 2023 Dec 21;18(12):e0296157. doi: 10.1371/journal.pone.0296157 (PMC10734938; doi:10.1371/journal.pone.0296157)

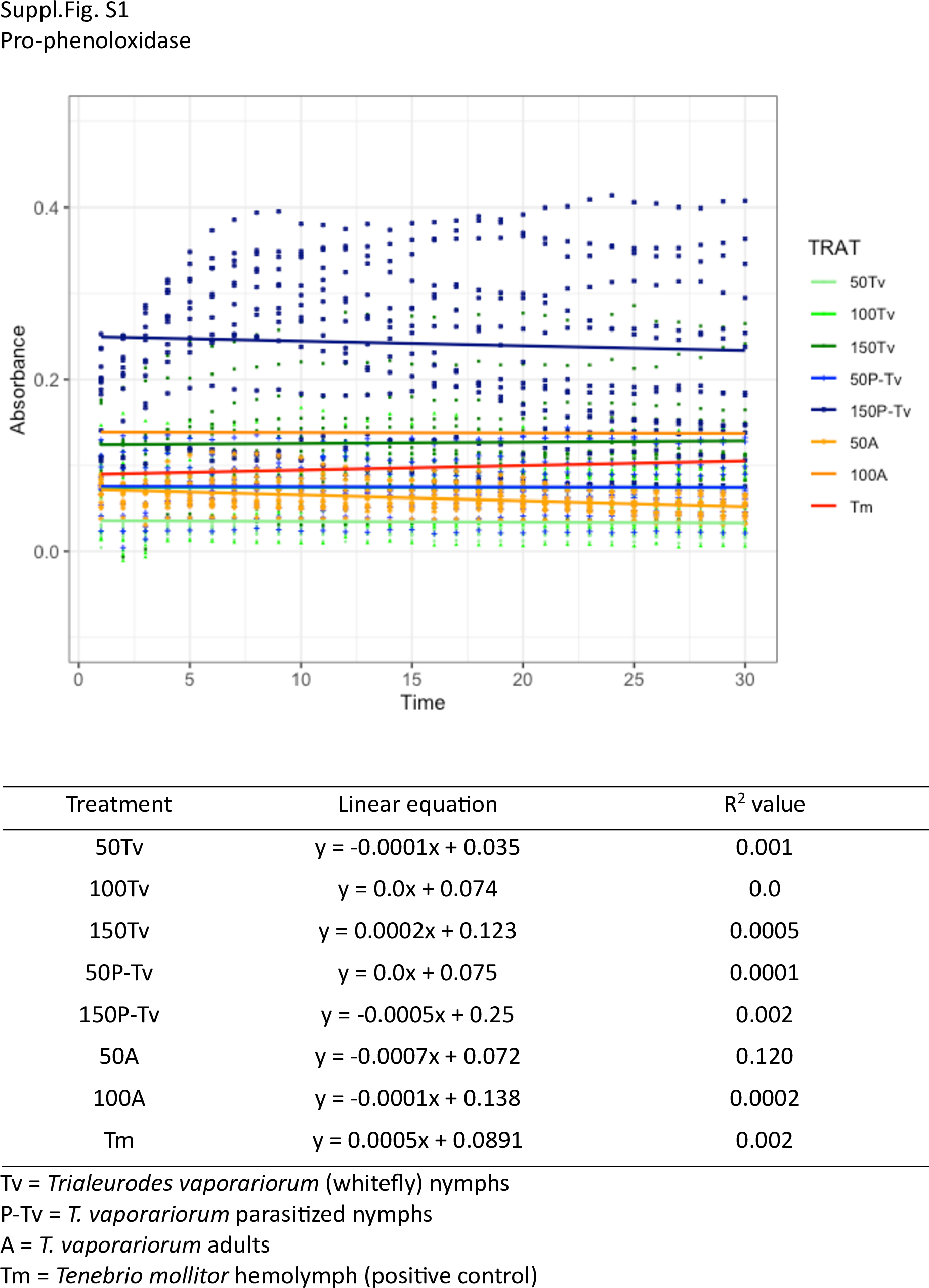

Supplement: S1 Fig — Top: absorbance levels of activated prophenoloxidase (proPO) in parasitized and non-parasitized nymphs and adults of the whitefly T. vaporariorum. Bottom: estimated linear equation and R2 value for each treatment, description of abbreviations is also provided. (TIF) [file pone.0296157.s001.tif]

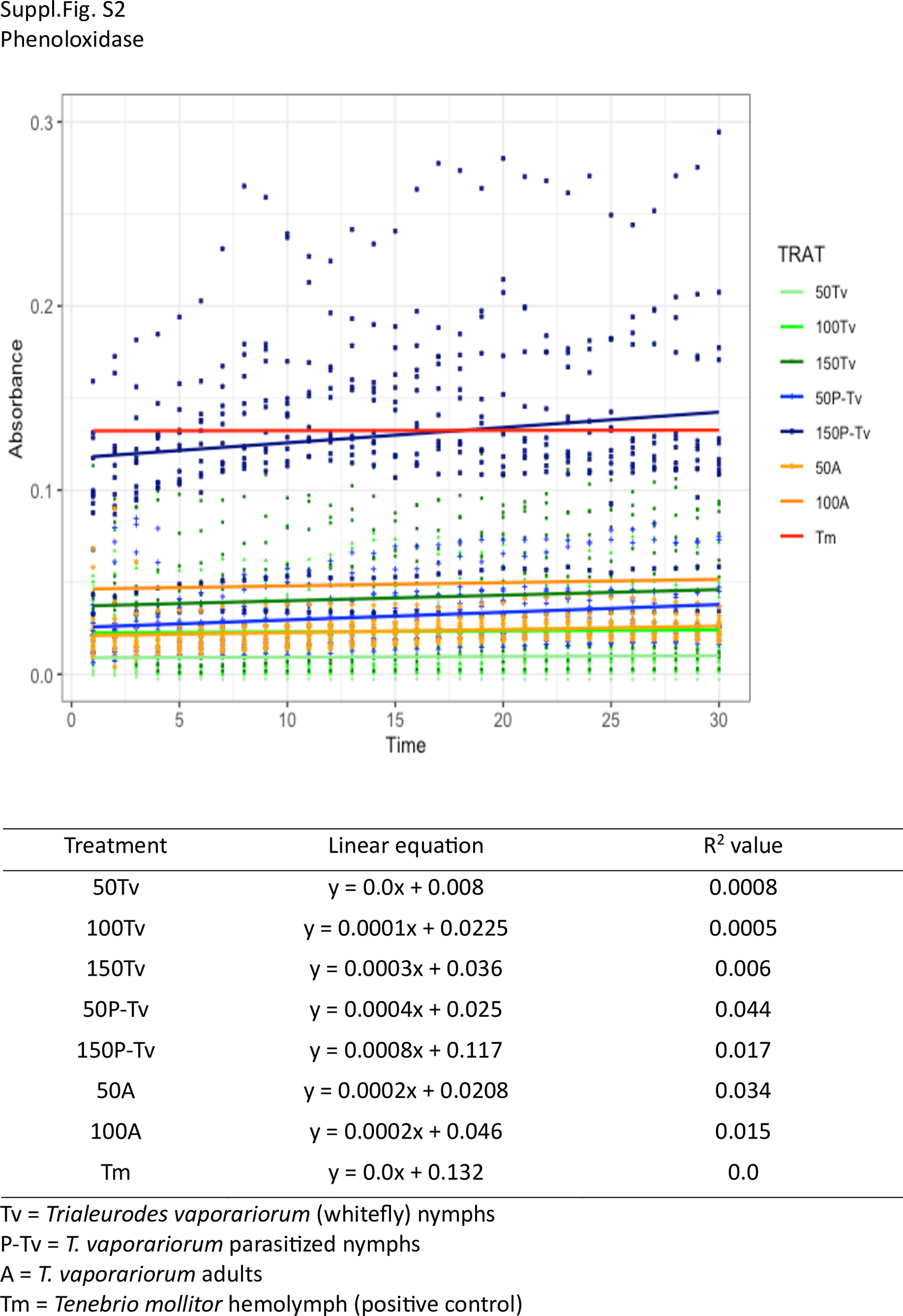

Supplement: S2 Fig — Top: absorbance levels of the enzymatic activity of phenoloxidase (PO) in parasitized and non-parasitized nymphs and adults of the whitefly T. vaporariorum. Bottom: estimated linear equation and R2 value for each treatment, description of abbreviations is also provided. (TIF) [file pone.0296157.s002.tif]

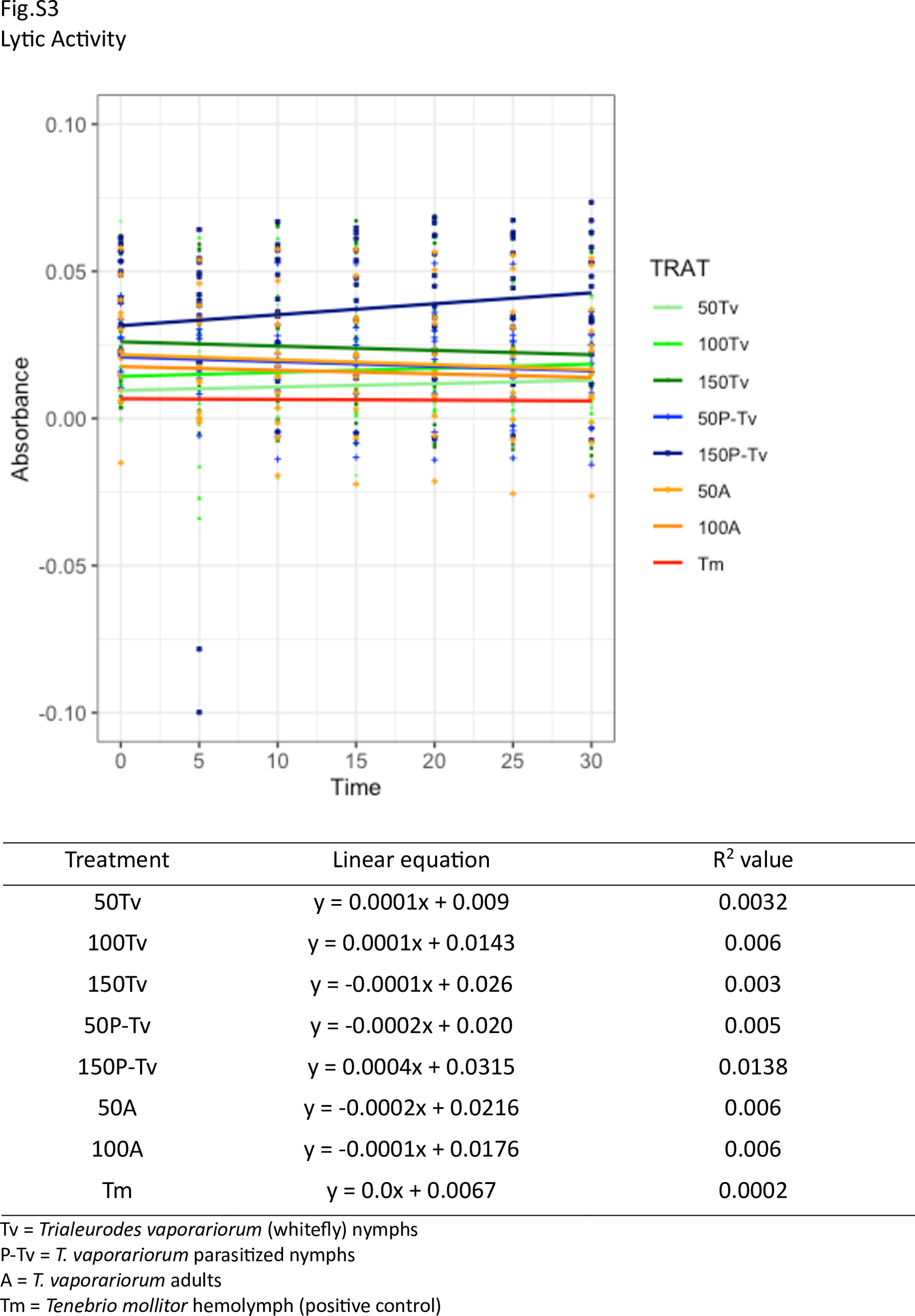

Supplement: S3 Fig — Top: absorbance levels of lytic activity (LA) in parasitized and non-parasitized nymphs and adults of the whitefly T. vaporariorum. Bottom: estimated linear equation and R2 value for each treatment, description of abbreviations is also provided. (TIF) [file pone.0296157.s003.tif]
